# Copper as Dietary Supplement for Bone Metabolism: A Review

**DOI:** 10.3390/nu13072246

**Published:** 2021-06-29

**Authors:** Mariangela Rondanelli, Milena Anna Faliva, Vittoria Infantino, Clara Gasparri, Giancarlo Iannello, Simone Perna, Antonella Riva, Giovanna Petrangolini, Alice Tartara, Gabriella Peroni

**Affiliations:** 1 IRCCS Mondino Foundation, 27100 Pavia, Italy; mariangela.rondanelli@unipv.it; 2 Department of Public Health, Experimental and Forensic Medicine, University of Pavia, 27100 Pavia, Italy; viriainfantino@hotmail.it; 3 Endocrinology and Nutrition Unit, Azienda di Servizi alla Persona ‘‘Istituto Santa Margherita’’, University of Pavia, 27100 Pavia, Italy; milena.faliva@gmail.com (M.A.F.); clara.gasparri01@universitadipavia.it (C.G.); alice.tartara01@universitadipavia.it (A.T.); 4 General Management, Azienda di Servizi alla Persona “Istituto Santa Margherita”, 27100 Pavia, Italy; direttoregenerale@asppavia.it; 5 Department of Biology, College of Science, University of Bahrain, Sakhir 32038, Bahrain; simoneperna@hotmail.it; 6 Research and Development Department, Indena SpA, 20139 Milan, Italy; antonella.riva@indena.com (A.R.); giovanna.petrangolini@indena.com (G.P.)

**Keywords:** copper, bone health, humans, supplementation, bone mineral density

## Abstract

While in vitro and animal studies of osteoblastic and osteoclastic activity as well as bone resistance for copper are numerous, and the results encouraging in terms of regulation, human studies are scarce. The aim of this narrative review was to investigate the correlation of blood copper, daily copper intake, and copper supplementation with bone mineral density. This review included 10 eligible studies: five studies concerned copper blood levels, one study concerned daily copper intake, and four studies concerned copper supplementation. Blood copper levels did not show statistically significant differences in four of the studies analyzed, while only one study showed differences between osteoporotic and healthy women, although only with women between 45 and 59 years of age and not between 60 and 80 years of age. The dietary copper intake among women with or without osteoporosis did not show any differences. Only one study with a small sample of subjects carried out these assessments; therefore, it is a topic that the literature must deepen with further studies. The two studies that analyzed the integration of copper (2.5–3 mg/day) only showed good results in terms of slowing down bone mineral loss and reducing resorption markers, confirming the effectiveness of copper supplementation on bone metabolism.

## 1. Introduction

In our organism there are between 50 and 120 mg of copper; almost two thirds of the body’s copper content is found in the muscles and skeleton, but the liver is also a key site for maintaining copper concentrations in plasma [1,2].

The quantity of copper absorption varies with dietary copper intake, ranging from over 50 percent at 1 mg/day to less than 20 percent at above 5 mg/day [3]. Part of the absorption occurs in the stomach where the acidic environment promotes the solubility of copper by dissociation from copper-containing macromolecules derived from food sources [4,5].

Turnover is faster and more copper is expelled into the gastrointestinal tract when there is more absorption. Excretion is probably the main point of regulation of the body’s total copper, and this efficient homeostatic regulation of absorption and retention helps to protect the body against copper toxicity and deficiency [3], while also considering that the excretory capacity of the kidney is limited [5]. The absorption of copper is inhibited by the presence of phytates, calcium, ascorbic acid, and other trace elements such as zinc, whose metabolism is linked to that of copper [6]. Indeed, it has been found that zinc supplementation in subjects with sickle cell anemia causes a deficiency of copper in the blood [7,8].

The RDA for adult women and men is 900 μg/day [9], and the foods with the highest content of copper are: bovine and ovine liver, dried fruit especially cashews, oysters, arigas, octopus, prawns, cod, mackerel, quail, and chicken and turkey leg [10].

A study carried out on healthy young people established that 0.38 mg of copper per day was not sufficient to maintain its balance in the body and that the minimum requirement should be between 0.4 and 0.8 mg/day [11].

The main function of copper is the constitution of enzymes that transfer electrons (oxidases) to achieve the reduction of molecular oxygen, and is therefore essential for energy metabolism at the cellular level [4,12,13]. Among these enzymes we find lysyl oxidase, which uses lysine and hydroxylysine (present in collagen and elastin) as substrates to produce the cross-links necessary for the development of connective tissues, including those of bones [9,14,15,16,17].

Various in vitro studies have ascertained the positive effect of copper on the cells that regulate bone metabolism; Li et al. showed that copper ion can inhibit osteoclastic resorption, [18] while other authors demonstrated that the positive effects of copper are dose-dependent. In particular, low concentrations (i.e., 0.1% *w*/*w*) of Cu improved the viability and growth of osteoblastic cells, while higher concentrations of Cu (i.e., 2.5% and 1% (*w*/*w*)) proved to be cytotoxic [19].

Furthermore, the presence of copper stimulates the differentiation of mesenchymal stem cells towards the osteogenic lineage [20].

Animal studies have shown that dietary copper deficiency can lead to a decrease in the copper present in our body and consequently to the reduction of the activity of some enzymes [5,21]. The physiological consequences of copper deficiency include connective tissue defects, which lead, among others, to skeletal problems [5].

A copper-deficient diet in chicks showed that this deficiency is able to block the formation of cross bonds in collagen and elastin from various tissues, with consequent bone fragility related to the nutritional deficiency of copper [22].

To evaluate bone strength in copper deficiency, a diet deficient in this mineral was administered to mice, which showed a reduction in mechanical strength. This situation was subsequently associated with defects in the bone collagen component [23].

A recent review summarized all these mechanisms of copper and its skeletal impact [24].

While in vitro and animal studies of osteoblastic and osteoclastic activity and bone resistance are numerous and the results encouraging in terms of regulation, human studies are scarce, and no reviews have been published on this topic. Therefore, the aim of this narrative review was to consider the correlation of blood copper, daily copper intake, and copper supplementation with bone mineral density.

## 2. Results

### 2.1. Blood Copper Concentrations in Relation to Bone Metabolism

This research was conducted with these keywords: “copper” AND “copper blood concentrations” AND “bone” AND “humans”. We analyzed a total of five studies: four cross-sectional studies and one case-control study.

The description of the studies are presented in Table 1.

### 2.2. Copper Intake in Relation to Bone Metabolism

This research was conducted with these keywords: “copper” AND “copper intake” AND “bone” AND “humans”. We analyzed a total of one cross-sectional study.

The description of the studies are presented in Table 2.

### 2.3. Copper Supplementation in Relation to Bone Metabolism

This research was conducted with these keywords: “copper” and “copper supplementation” and “bone” and “humans”. A total of four studies were analyzed: two double-blind placebo-controlled trial, one double-blind randomized trial, and one longitudinal study.

The description of the studies are presented in Table 3.

## 3. Discussion

A total of five studies evaluated the blood levels of copper in osteoporotic, osteopenic, and healthy subjects. While there were no statistically significant differences in copper blood levels between the population groups investigated in four of the papers [26,27,28,29], outcomes were the opposite in Okyay’s 2013 study. A total of 728 menopausal, osteoporotic, or healthy women were enrolled and divided into age groups (45–59 years and 60–80 years); blood levels of copper and bone mineral density in different districts (lumbar L1–L4, neck of the femur, and whole femur) in these women were evaluated. The study results demonstrated a statistically significant difference in blood copper levels between osteoporotic and healthy women in all the districts analyzed, but only in the 45–59 age group [25].

A single 2015 study compared the dietary copper intake of postmenopausal women with osteopenia and osteoporosis, demonstrating the absence of a statistically significant difference between the two groups [28].

Regarding the studies that evaluated copper supplementation in relation to bone metabolism, the individual effects of copper were assessed in two studies [32,33], while two other papers evaluated the effects of a micronutrient association [30,31].

The Eaton-Evans study evaluated the effects of supplementation with 3 mg of copper versus placebo, in a group of healthy women, for a total of 2 years, subsequently coming to the conclusion that there seems to be a reduction in the loss of vertebral bone mineral density in the treated group [32].

A different type of study was instead designed by Baker et al. A total of 11 healthy men were identified and given several levels of copper intakes. The subjects all followed a 3-week diet that started with medium copper content (1.6 mg/d), followed by low copper content (0.7 mg/d), and lastly, with high copper content (6 mg/d). The basic power supply was always that which corresponded to the low copper intake, with different additions of copper sulfate to achieve the average intake or the high intake. Between the first and the second period, i.e., the transition from medium to low copper intake, an increase in some bone resorption markers was assessed, while these markers significantly decreased with the transition from the deficient to the rich copper diet [33].

The remaining two papers have conflicting conclusions. Nielsen’s 2011 study, which compared calcium supplementation versus calcium supplementation associated with copper and zinc in a group of postmenopausal women emphasized the effectiveness of the association of zinc but not copper [30]. However, in the study by Strause, healthy postmenopausal women were involved and divided into four groups (calcium supplement + micronutrient supplement, calcium supplement + micronutrient placebo, calcium placebo + micronutrient supplement, placebo of calcium + placebo of micronutrients), where the group that received both calcium and micronutrient supplements, including copper, maintained lumbar bone mineral density with a significant difference as compared to the group that received only placebo. The remaining two groups were at an intermediate level, demonstrating no significant differences with the treated group or with the placebo group [31].

## 4. Materials and Methods

This narrative review was performed following Egger et al. [34] with these steps:

1. Configuration of the working group: two operators, experts in clinical nutrition (one acting as a methodological operator and one participating as a clinical operator).
2. Formulation of the questions based on the considerations indicated in the abstract: the correlation of blood copper, daily copper intake, and copper supplementation with bone mineral density.
3. Recognition of the relevant studies. Research was carried out on PubMed (Public Medline run by the National Center of Biotechnology Information (NCBI) of the National Library of Medicine of Bethesda (USA)) as follows: (a) definition of the keywords (copper, humans, bone health, bone mineral density, supplementation), inserting the interest field of the documents to be searched, grouped in quotation marks (“…”), and used separately or in combination; (b) use of the Boolean variable (true or false) AND operator, that allows for the establishment of logical relations among concepts; (c) research modalities: advanced search; (d) limits: papers published in the last 20 years; humans; adults; languages: English; (e) manual search performed by the researchers experienced in clinical nutrition through the revision of articles, focusing on the effectiveness of copper supplementation (alone or with other micronutrients) on the growth and maintenance of bone in humans, in order to suggest a daily dosage for copper supplementation.
4. Published in journals qualified in the Index Medicus.
5. Analysis and presentation of the outcomes: paragraphs about effectiveness of copper supplementation alone or in combination with other nutrients were created, and the data extrapolated from the “revised studies” were collocated in tables; in particular, the author, year of publication, and the characteristics of the study were specified for each study.
6. An analysis of the reports in the form of a narrative review was carried out. At the beginning of each section, the type of studies chosen and the keywords considered are reported. Studies of any design which considered the effectiveness of copper supplementation (alone or with other micronutrients) on the growth and maintenance of bone in humans were evaluated.

**Figure 1:**
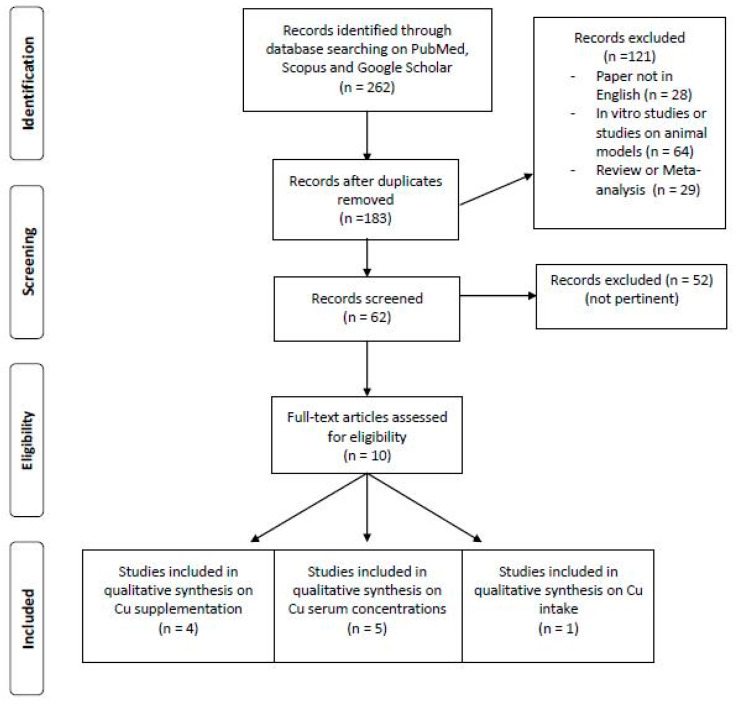
Flow chart for research of the literature.

Figure 1 indicates the flow chart for the research of the literature.

## 5. Conclusions

While in vitro and animal studies of osteoblastic and osteoclastic activity and bone resistance are numerous and the results encouraging in terms of regulation, human studies are scarce.

Blood copper levels did not show statistically significant differences in four of the studies analyzed, while only one study showed differences between osteoporotic and healthy women, although only with women 45–59 years of age and not 60–80 years of age.

The dietary copper intake among women with or without osteoporosis did not show any differences. Only one study with a small sample of subjects carried out these assessments; therefore, it is a topic that the literature must deepen with further studies.

The two studies that analyzed the integration of copper (2.5–3 mg/day) only showed good results in terms of slowing down bone mineral loss and reducing resorption markers, thus confirming their effectiveness.

## Figures and Tables

**Table 1:** Studies that considered blood copper levels.

First Author, Year	Study Design	Institution and Country	Inclusion Criteria	Number of Subjects (M-F) Mean Age	Micronutrient Serum Concentration Osteoporosis	Micronutrient Serum Concentration Osteopenia	Micronutrient Serum Concentration Normal	Primary Outcomes	Results
Okyay E, 2013 [25]	Cross-sectional study	Dokuz Eylul University School of Medicine, Izmir, Turkey	Postmenopausal women between age 45 and 80	728 F 56.52 ± 6.14 y	Women at 45–59 years: (*p* value < 0.05) Cu (μg/mL): - L1–L4 osteoporotic (OP) 96.6 ± 39 - Total femur OP 90 ± 37.5 - Femoral neck OP 96.3 ± 33.9		Women at 45–59 years: (*p* value < 0.05) CU (μg/mL): - L1–L4 non OP 112.6 ± 28.2 - Total femur non OP 111.3 ± 30.2 - Femoral neck non OP 111.4 ± 30.9	Relationship between serum main minerals and postmenopausal osteoporosis.	Low Cu serum levels were independent risk factors for development of OP especially in early menopausal period.
Mutlu M, 2007 [26]	Cross-sectional study	Erciyes University, Kayseri, Turkey	Post-menopausal women	120 F 40 OP 58 ± 8.40 y, osteopenic 57 ± 9 y and 40 healthy group 59 ± 6 y	Cu (mg/L) 1.54 ± 0.12	Cu (mg/L) 1.59 ± 0.09	Cu (mg/L) 1.60 ± 0.08	Cu changes in osteoporotic, osteopenic, and normal postmenopausal women.	No statistically significant differences observed between the osteopenic, osteoporotic, and control groups with respect to copper levels.
Arikan DC 2011 [27]	Case–control study	Medical Faculty of Kahramanmaras Sutcu Imam (Kahramanmaras, Turkey)	Natural menopause for more than 6 months	107 F 35 healthy 48.17 ± 7.78 y, 37 osteopenic 55.32 ± 7.78 y and 35 OP 60.57 ± 8.65 y	Cu (μg/dL) 138.76 ± 37.21	Cu (μg/dL) 137.58 ± 31.73	Cu (μg/dL) 140.92 ± 32.74	Serum Cu levels in postmenopausal women with osteoporosis, osteopenia, and in healthy controls, and the relationship between Cu and bone mineral density (BMD).	Plasma levels of all parameters were similar across each group (*p* > 0.05).
Mahdavi-Roshan, 2015 [28]	Cross-sectional study	Rheumatology clinic in Tabriz, Islamic Republic of Iran	women > 50 years old postmenopausal, having no history of hormone replacement therapy	51 F 57.97 ± 1.2 y		Serum Cu (μg/dL): 27.29 ± 1.26	Serum Cu (μg/dL) 26.75 ± 1.35	Investigate and compare the mineral status between osteopenic and osteoporotic postmenopausal women.	No statistically significant differences between the osteopenic and osteoporotic groups with respect to serum levels of Cu.
LIU SZ, 2009 [29]	Cross- sectional study	Xi’an urban area, China	45 to 65-year-old females in natural and no hormone drug intake and osteoporosis treatment 6 months before investigation	290 F 54.4 ± 5.5 y	Cu serum (mg/L) 0.8873 ± 0.2930	Cu serum (mg/L) 0.8528 ± 0.2397	Cu serum (mg/L) 0.8498 ± 0.3106	Correlation between serum macroelement and trace element contents and BMD as well as the occurrence of osteoporosis.	There exist significant correlations between the contents of serum Cu, but no significant differences in this element content between the osteoporosis group, osteopenia group, and healthy group.

**Table 2:** Studies that considered copper intake.

First Author, Year	Study Design	Institution and Country	Inclusion Criteria	Number of Subjects (M-F) Mean Age	Lowest Quintile Intake/RDA or EAR	% Subject in Lowest Quintile Intake/% Subject < RDA or EAR	Highest Quintile Intake	% Subject in Highest Quintile Intake	Primary Outcomes	Results
Mahdavi-Roshan, 2015 [28]	Cross-sectional study	Rheumatology clinic in Tabriz, Islamic Republic of Iran	Postmenopausal women > 50 years, having no history of hormone replacement therapy	51 F 57.97 ± 1.2 y	The mean dietary intake (and percent from RDA) of copper in post-menopausal women with low bone density was 1.07 ± 0.08 mg/day (120 ± 12.2% RDA)				Investigate and compare the mineral status between osteopenic and osteoporotic postmenopausal women.	No statistically significant differences between the osteopenic and osteoporotic groups with respect to dietary intake copper.

**Table 3:** Studies that considered copper supplementation.

First Author, Year	Study Design	Institution and Country	Inclusion Criteria	Intervention	Parallel Treatments	Number of Subjects (M-F) Mean Age	Duration of the Intervention	Primary Outcomes	Results
Nielsen FH, 2011 [30]	double-blind, placebo-controlled design.	University of North Dakota	postmenopausal women aged 51–80 y, BMI ≤ 32 kg/m^2^, BMD not more than 2·5 standard deviations below that for young adults	600 mg calcium (Ca) supplement plus a 2 mg Cu (copper gluconate) and 12 mg Zn (zinc gluconate) supplement.	supplement containing 600 mg Ca plus a maize starch placebo	649 F	2 years	How Cu and Zn intakes would reduce the risk for bone loss.	Cu supplementation apparently did not have an impact on whole-body bone contents, BMD.
Strause L, 1994 [31]	double-blind, placebo-controlled trial.	San Diego greater Metropolitan area	>50 y old and in good general health	(1) placebo Ca + active trace minerals, (2) active Ca + placebo trace minerals, (3) active Ca + active trace mineral: 1000 mg elemental calcium/d in the form of Ca citrate malate and active supplement contained 15.0 mg of Zn as sulfate salt, 2.5 mg of Cu, and 5.0 mg of manganese as gluconate salts.	placebo calcium + placebo trace minerals	59 F 66 ± 7 y	2 years	Impact of supplementary Ca with and without the addition of a combination trace elements on spinal bone loss.	Supplementation with 1000 mg of Ca, 15 mg of Zn, 5 mg of manganese, and 2.5 mg of Cu maintained spinal bone density and differed significantly from a placebo group that lost bone density.
Eaton-Evans 2003 [32]	random and double-blind study	Royal Victoria Hospital, north Belfast	healthy women, aged 45–56 years	3 mg Cu as amino acid chelate	placebo	73 F Cu group: 49.97± 3.1 Placebo group: 50.8 ± 3.5	2 years	Effects of Cu supplementation over 2 years on vertebral trabecular bone mineral density (VTBMD).	Cu supplementation appeared to have reduced the loss of VTBMD in these middle-aged women over a 2-year period.
Baker A 1999 [33]	longitudinal intervention trial	Institute of Food Research, Norwich, UK	Subjects without any history of bone or articular disease, and with no intake of medicine that could affect bone or cartilage metabolism	Medium (1.6 mg/d), low (0.7 mg/d) and high (6.0 mg/d) intakes of Cu, in that order. A 7 d rotating low Cu menu was formulated and analysed for Cu content. This low Cu diet (0.7 mg/d) was fed throughout the three dietary periods and was supplemented to the appropriate level of Cu (as a CuSO_4_ solution dissolved in de-mineralised water taken with a meal) to achieve the medium (1.6 mg/d) and high (6.0 mg/d) Cu intakes.	//////////	11 M 30.9 y	8-week dietary periods with a minimum of 4-week washout periods.	Effects of changing from a medium (1.6 mg Cu/d) to a low (0.7 mg Cu/d) or a high (6.0 mg/d) Cu intake on biochemical indices of bone turnover in healthy adult males.	Biomarkers of bone resorption were significantly increased when subjects were switched from the medium to the low Cu diet.

## Data Availability

The data presented in this study are available in this article.
